# Two-Dimensional Transition Metal Dichalcogenide: Synthesis, Characterization, and Application in Candlelight OLED

**DOI:** 10.3390/molecules30010027

**Published:** 2024-12-25

**Authors:** Dipanshu Sharma, Sanna Gull, Anbalagan Ramakrishnan, Sushanta Lenka, Anil Kumar, Krishan Kumar, Pin-Kuan Lin, Ching-Wu Wang, Sinn-Wen Chen, Saulius Grigalevicius, Jwo-Huei Jou

**Affiliations:** 1 Department of Materials Science and Engineering, National Tsing Hua University, 101, Sec. 2, Guang-Fu Road, Hsinchu 30013, Taiwan; dipanshusharma7374@gmail.com (D.S.); sannagull@gapp.nthu.edu.tw (S.G.); sushantalenka1@gmail.com (S.L.); anilgpchkee@gmail.com (A.K.); kuan841216@gmail.com (P.-K.L.); 2 Department of Chemical Engineering, National Tsing Hua University, 101, Sec. 2, Guang-Fu Road, Hsinchu 30013, Taiwan; anbusrr@gmail.com (A.R.); swchen@mx.nthu.edu.tw (S.-W.C.); 3 School of Chemical Sciences Indian Institute of Technology, Mandi 175005, Himachal Pradesh, India; krishanme906@gmail.com; 4 Graduate Institute of Opto-Mechatronics, Department of Mechanical Engineering, National Chung Cheng University, Chiayi County 62102, Taiwan; melcww@ccu.edu.tw; 5 Department of Polymer Chemistry and Technology, Kaunas University of Technology, Radvilenu Plentas 19, LT50254 Kaunas, Lithuania

**Keywords:** candlelight OLEDs, TMDs, ESG goals, color temperature, HIL

## Abstract

Low-color-temperature candlelight organic light-emitting diodes (OLEDs) offer a healthier lighting alternative by minimizing blue light exposure, which is known to disrupt circadian rhythms, suppress melatonin, and potentially harm the retina with prolonged use. In this study, we explore the integration of transition metal dichalcogenides (TMDs), specifically molybdenum disulfide (MoS_2_) and tungsten disulfide (WS_2_), into the hole injection layers (HILs) of OLEDs to enhance their performance. The TMDs, which are known for their superior carrier mobility, optical properties, and 2D layered structure, were doped at levels of 0%, 5%, 10%, and 15% in PEDOT:PSS-based HILs. Our findings reveal that OLEDs doped with 10% MoS_2_ exhibit notable enhancements in power efficacy (PE), current efficacy (CE), and external quantum efficiency (EQE) of approximately 39%, 21%, and 40%, respectively. In comparison, OLEDs incorporating 10% of WS_2_ achieve a PE of 28%, a CE of 20%, and an EQE of 35%. The enhanced performance of the MoS_2_-doped devices is attributed to their superior hole injection and balanced carrier transport properties, resulting in more efficient operation. These results highlight the potential of incorporating 2D TMDs, especially MoS_2_, into OLED technology as a promising strategy to enhance energy efficiency. This approach aligns with environmental, social, and governance (ESG) goals by emphasizing reduced environmental impact and promoting ethical practices in technology development. The improved performance metrics of these TMD-doped OLEDs suggest a viable path towards creating more energy-efficient and health-conscious lighting solutions.

## 1. Introduction

High-color-temperature white light sources emit a significant amount of blue light, which has raised significant concerns due to its adverse health and environmental effects. Exposure to blue light is known to disrupt circadian rhythms, potentially cause retinal damage, and increase the risk of diseases such as insomnia, melatonin suppression, obesity, and cancer [1,2,3]. In addition, blue light pollution can damage ecosystems and lead to the deterioration of cultural artifacts [4,5]. These issues underscore the urgent need for advanced lighting technologies that mitigate blue light exposure while maintaining high efficiency and performance. On the other hand, 2D transition metal dichalcogenides (TMDs) have garnered significant interest due to their remarkable electronic, optical, and mechanical properties. These materials have a tunable bandgap that transitions from an indirect to a direct configuration upon exfoliation [1,4,5], which significantly enhances their suitability for hole transfer in optoelectronic applications [6,7]. Several studies have highlighted the significance of this bandgap transition in applications such as photodetectors and solar cells [8,9]. In addition, TMDs exhibit remarkable stability and photoluminescence efficiency [10], contributing to improved light absorption and emission in devices such as organic light-emitting diodes (OLEDs) [11,12]. Recent studies indicate that TMDs can be engineered to improve charge transport properties, further enhancing device performance [13,14,15,16]. However, device efficiency remains a key challenge for the industrial application of OLEDs. To address this issue, it is essential to optimize charge injection and transport while ensuring effective exciton confinement within the emitting layer. In this context, the anode, typically made of indium-tin oxide (ITO), facilitates efficient hole injection due to its favorable work function, which is well matched to that of PEDOT:PSS. Conversely, the work function of the cathode is well matched to the electron injection layer, lithium fluoride (LiF). As a result, holes and electrons efficiently migrate to the emitter layer, where they recombine to form excitons [17]. These factors are crucial for achieving high performance, as highlighted by Lee et al. (1999), Wang et al. (2008), and Song et al. (2018) [18,19,20]. Lee et al. (1999) utilized ultraviolet photoemission spectroscopy to analyze electronic properties at metal–organic and organic–organic interfaces, revealing that while vacuum-level alignment does not apply to metal–organic interfaces, it holds true for organic–organic interfaces, which influences device efficiency. Wang et al. (2008) demonstrated that a hole-injection barrier in trilayer OLEDs enhances current efficiency by optimizing hole transport, reducing recombination losses. Song et al. (2018) developed all-solution-processed OLEDs with a hybrid electron injection layer that improves electron injection and solvent resistance, leading to higher luminous efficiency. Building on these insights, recent work by Zhao et al. (2023) [21] explored planar heterojunction (PHJ) OLEDs, uncovering competitions between exciton and exciplex emissions and identifying a Dexter energy transfer channel that impacts design strategies for high-performance white OLEDs. Furthermore, Xie et al. (2024) [22] introduced a novel approach to improve charge balance by optimizing both the hole injection layer and electron transporting layer, demonstrating significant enhancements in external quantum efficiency and device lifetime. The incorporation of TMDs can play a significant role in overcoming these challenges, thereby improving the overall efficiency of OLEDs. Furthermore, previous studies have demonstrated the effectiveness of TMDs in enhancing OLED performance. For example, the incorporation of MoS_2_ into PEDOT:PSS as a hole injection layer has led to a remarkable 28% increase in current efficiency for green phosphorescent organic light-emitting diodes (OLEDs) [23], significantly optimizing charge injection and overall device performance. Doping PEDOT:PSS with solution-processed WS_2_ enhances hole injection in near-ultraviolet OLEDs [24], resulting in a maximum radiance of 4.7 mW/cm^2^ and an external quantum efficiency of 2.1%. Besides, the use of WS_2_ contributes to improved film morphology and electronic properties, allowing for better carrier balance and increased device efficiency. By incorporating TMDs into OLEDs, particularly in the hole injection layers, we aim to optimize device performance while also addressing the critical health implications of blue light exposure. This approach not only seeks to improve energy efficiency and lighting quality but also aligns with environmental, social, and governance (ESG) goals. Our findings underscore the potential of TMD-doped OLEDs as a solution for developing healthier, more sustainable lighting technologies.

In this study, we investigate the incorporation of 2D TMDs into OLEDs to enhance their performance in candlelight applications. Specifically, we investigate the effect of doping hole injection layers (HILs) with MoS_2_ and WS_2_ on key performance metrics. Our results indicate that OLEDs doped with 10% MoS_2_ achieve significant performance increments of approximately 39%, 21%, and 40% in PE, CE, and EQE, respectively. Conversely, OLEDs doped with 10% WS_2_ exhibit a PE of 28%, CE of 20%, and an EQE of 35%. These results highlight the potential of MoS_2_-doped OLEDs as an effective solution for the development of energy-efficient and health-conscious lighting technologies.

## 2. Results and Discussion

**Figure 1:**
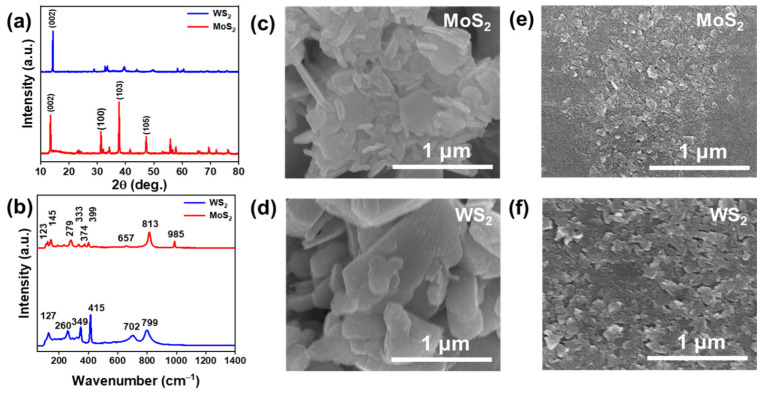
Structural and morphological characterization of MoS_2_ and WS_2_. (**a**) PXRD patterns show characteristic diffraction peaks for (bulk) MoS_2_ and WS_2_. (**b**) Raman spectra provide insight into the structural properties of both materials (bulk). SEM images show the morphology of (**c**) bulk and (**e**) exfoliated MoS_2_, highlighting the transformation into smaller nanosheets. (**d**,**f**) show the corresponding SEM images for bulk and exfoliated WS_2_, respectively.

The crystal structural analysis of the as-synthesized catalysts was performed using powder X-ray diffraction (PXRD). The PXRD patterns of MoS_2_ and WS_2_ are depicted in Figure 1a. In particular, MoS_2_ exhibits characteristic diffraction peaks at 2θ values of 13.4°, 31.3°, 37.8°, 47.2°, 55.9°, 57.7°, 69.4°, 72.1°, and 76.2° corresponding to the (002), (100), (103), (105), (110), (008), (108), (208), and (116) diffraction planes of MoS_2_ (JCPDS No: 06-0097), respectively [25]. The highly intense characteristic peak of WS_2_ (JCPDS No. 08-0237) is present at 14.3°, which corresponds to the (002) plane [26]. These PXRD patterns illustrate the distinct crystalline phases and structural features of MoS_2_ and WS_2_, confirming the quality and purity of the synthesized materials through their characteristic lattice spacings. Raman spectroscopy was also performed on MoS_2_ and WS_2_, and the results are shown in Figure 1b. The Raman spectra for WS_2_ show several peaks, including one at 127 cm^−1^ corresponding to the LA(M) mode, and peaks at 349 cm^−1^ (E_2g_) and 415 cm^−1^ (A_1g_), which are in good agreement with the previously reported literature [27]. The Raman measurements of MoS_2_ show similar spectral features, including the E_2g_ and A_1g_ vibrational modes, confirming the structural properties and quality of both synthesized materials [28]. The morphological analysis of the as-synthesized MoS_2_ and WS_2_ catalysts was performed by SEM, and the results are shown in Figure 1c,e for MoS_2_ and Figure 1d,f for WS_2_. The SEM images show clear structural differences between bulk and exfoliated samples. For MoS_2_, the bulk powder shows an agglomerated sheet-like structure, while the exfoliated MoS_2_ shows smaller, more uniform nanostructures. Similarly, bulk WS_2_ exhibits large agglomerated sheets that are reduced to smaller sheets after exfoliation, demonstrating the effectiveness of the exfoliation process in achieving layered nanostructures.

The RMS (root mean square) value in AFM is a key parameter used to quantify the surface roughness of a sample. It is a statistical measure that reflects the average deviation of surface heights from the mean plane, offering insights into the texture and topographical variations of the surface. Specifically, in the context of surface roughness, the RMS value is referred to as R_q_ (root mean square roughness), which is the standard term used for this measurement in AFM. A higher R_q_ value indicates a rougher surface, while a lower R_q_ value suggests a smoother, more uniform surface. This metric helps characterize the quality and consistency of materials in various applications. Mathematically, the RMS roughness is calculated as:

(1) $$R_{q}=\sqrt{\frac{1}{N}\sum_{i=1}^{n}{\left(z_{i}-z_{mean}\right)}^{2}}$$

where *z_i_* is the height at each point on the surface, *Z_mean_* is the mean height of the surface, and *N* is the total number of data points. The data presented in Table 1 show that MoS_2_ samples exhibit superior performance compared to WS_2_ samples. MoS_2_ exhibits better R_q_ roughness values, indicating a smoother surface, which is advantageous for the intended application. For example, at 5% doping concentration, MoS_2_ has an R_a_ (arithmetic average roughness) of 0.438 nm and an R_q_ of 0.558 nm, significantly lower than the corresponding values for WS_2_ (R_a_: 0.619 nm, R_q_: 0.801 nm). As the doping concentration increases to 10%, MoS_2_ maintains its advantageous roughness with an R_a_ of 0.410 nm and R_q_ of 0.523 nm, while WS_2_ shows only a slight improvement (R_a_: 0.483 nm, R_q_: 0.606 nm). At the highest doping concentration of 15%, MoS_2_ maintains a favorable R_a_ of 0.427 nm and R_q_ of 0.552 nm compared to WS_2_, which has an R_a_ of 0.532 nm and R_q_ of 0.669 nm.

PEDOT:PSS, however, has the highest R_q_ value (1.03 nm) among the series, suggesting it has the roughest surface compared to MoS_2_ and WS_2_. Despite this, the R_q_ value for PEDOT:PSS still indicates minimal height variations, reflecting a relatively flat surface. This smoothness is beneficial for various applications: in optoelectronic devices like solar cells and (light-emitting diodes) LEDs, it can reduce scattering and defects, enhancing electrical and optical performance; in thin-film coatings, it indicates uniform deposition, which is critical for precision-based applications; and in biocompatible coatings or biosensors, smoother surfaces can influence favorable interactions with biological materials.

**Figure 2:**
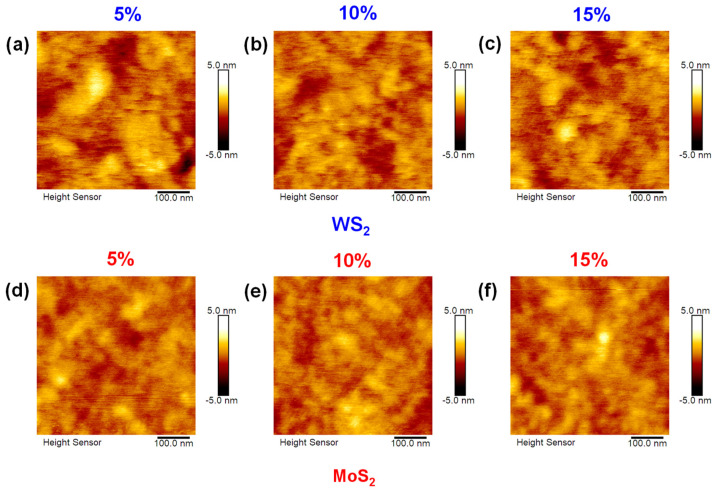
AFM images of WS_2_ (**a**–**c**) and MoS_2_ (**d**–**f**) doped in PEDOT:PSS at a concentration of 5%, 10%, and 15%, respectively.

The AFM images in Figure 2 illustrate the morphological differences between WS_2_ and MoS_2_ at different doping levels. Specifically, Figure 2a–c show the surface characteristics of WS_2_ at 5%, 10%, and 15% doping concentrations, respectively, to illustrate the effect of increasing doping on the surface morphology. In contrast, Figure 2d–f show the corresponding images for MoS_2_, demonstrating a consistent trend toward smoother surfaces with increasing doping levels.

This morphological analysis underscores the effectiveness of MoS_2_ as a dopant, which is consistent with the quantitative results in Table 1, and highlights its potential to improve the performance of optoelectronic devices and thin film coatings. In addition, UPS was utilized to determine the highest occupied molecular orbital (HOMO) energy levels of these materials, as shown in Figure S1. The HOMO level for WS_2_, depicted in Figure S1b, is found to be 4.8 eV, while MoS_2_, represented in Figure S1d, exhibits a HOMO level of 5.3 eV. To calculate the lowest unoccupied molecular orbital (LUMO) levels for both materials, the relationship $E_{LUMO}={-E}_{HOMO}+E_{g}$ [29,30,31,32] was applied, where $E_{g}$ is optical bandgap. For MoS_2_, this results in a LUMO of approximately 3.5 eV, while WS_2_ yields a LUMO of around 2.8 eV. For reference, the AFM image of the control device, pure PEDOT:PSS, is shown in Figure S2. The UV–vis spectroscopy analysis of MoS_2_ and WS_2_ nanostructures reveals significant optoelectronic properties, as illustrated in Figure S3. For WS_2_, Figure S3b indicates an optical bandgap of approximately 2 eV. In the case of MoS_2_, Figure S3d shows an optical bandgap of about 1.8 eV. These values highlight the potential of these materials for applications in optoelectronic devices. The compatibility of these energy levels underscores the suitability of MoS_2_ and WS_2_ nanostructures for use in advanced optoelectronic applications, facilitating efficient charge transport and injection.

### 2.1. Hole-Only Device (HOD)

**Figure 3:**
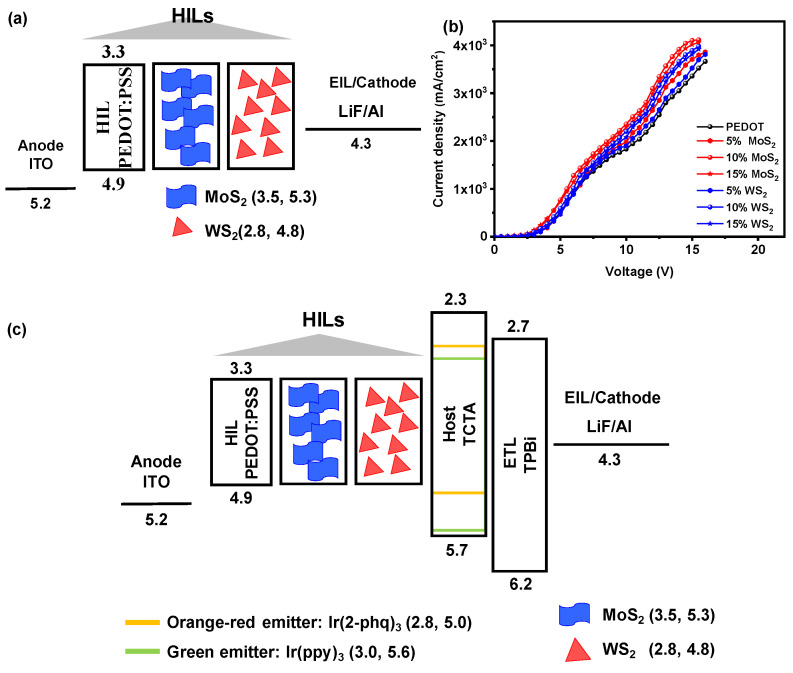
Performance of hole-only devices (HOD), (**a**) device structure, and (**b**) current density vs. voltage plot of different MoS_2_ and WS_2_ concentrations, showing the effect on hole transport properties. (**c**) Energy level diagram illustrating the alignment of energy levels in hybrid PEDOT:PSS, highlighting its impact on hole transport for OLED applications.

To investigate the current density behavior in HODs, we fabricated devices with a configuration similar to previous studies: ITO (~125 nm)/HIL (~35 nm)/LiF (~1 nm)/Al (~100 nm). The HIL was prepared by incorporating different concentrations of MoS_2_ and WS_2_ into a PEDOT:PSS solution. Our results showed that the addition of 10 wt% MoS_2_ in PEDOT:PSS resulted in the most favorable hole transport properties. This concentration showed the best current density performance among the variations tested. The experimental section describes the comprehensive fabrication process for these HODs. Figure 3a,b illustrate the structural layout and the current density vs. voltage relationship. Our comparative analysis shows that the incorporation of MoS_2_ significantly improves the hole injection efficiency, as evidenced by a noticeable shift in the current density curves. In particular, the devices with MoS_2_ exhibit higher current densities at lower voltages compared to those with WS_2_, indicating improved hole transport. Furthermore, the current density plots show that while both TMDs provide improvements over the undoped PEDOT:PSS device, MoS_2_ outperforms WS_2_ over the tested voltage range. This is attributed to the superior hole injection properties and optimized energy levels of MoS_2_ hole injection properties and optimized energy levels, which allow for more efficient charge transport. These results underscore the importance of selecting the appropriate dopant to achieve optimal device performance.

### 2.2. Device Performance and Characterization of Solution-Processed Phosphorescent OLEDs

In this study, the electroluminescence properties of candlelight devices were investigated, focusing on the effect of doping the hole injection layer (HIL) of PEDOT:PSS with different concentrations of MoS_2_ and WS_2_ on their performance characteristics. Figure 3c illustrates the energy level diagrams of the devices, highlighting the alignment of the HOMO and LUMO levels between the HIL and PEDOT:PSS. 

The alignment follows a ladder-like structure, which facilitates efficient hole injection by minimizing the hole injection barrier, a critical factor for charge transport into the active layer. MoS_2_, in particular, demonstrates an excellent alignment of its HOMO level with that of PEDOT:PSS, facilitating smoother hole transport and enhancing device performance. This alignment results in a significant reduction of energy barriers for hole injection, leading to higher device efficiencies. While WS_2_ also exhibits good alignment, MoS_2_ offers a slightly better match with the HOMO level of PEDOT:PSS, resulting in even more efficient hole injection and higher device performance. Table S1 presents the hole mobility data derived from space-charge-limited current (SCLC) measurements for devices with MoS_2_- and WS_2_-doped HILs, which further validate the impact of energy level alignment on reducing the hole injection barrier and enhancing device performance. These findings underscore the importance of dopant selection and energy level tuning in optimizing the performance of OLED devices, providing valuable insights into the design of more efficient hole injection layers. The methods and characterization techniques used for device fabrication are detailed in the experimental section. The performance of OLEDs doped with varying concentrations of MoS_2_ and WS_2_ was systematically evaluated against a PEDOT:PSS control device as shown in Table 2.

**Figure 4:**
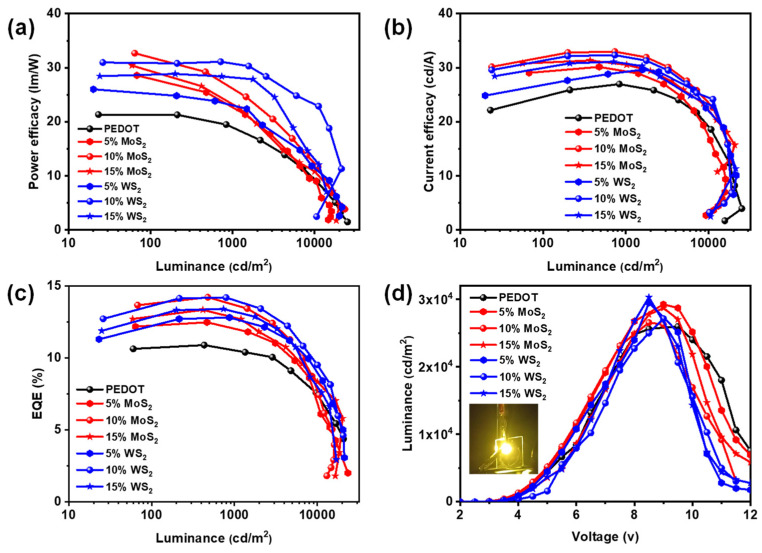
Effects of different hole injection layers (HILs) on the (**a**) power efficacy vs. luminance, (**b**) current efficacy vs. luminance, (**c**) EQE vs. luminance, and (**d**) luminance vs. voltage (inset: glowing device) of candlelight OLED devices.

This device achieved a PE of 23.6 lm/W, a CE of 25.7 cd/A, and an EQE of 9.7% at 100 cd/m^2^. In contrast, OLEDs with 10% MoS_2_ demonstrated a PE of 32.7 lm/W, a CE of 21 cd/A, and an EQE of 13.6%, representing enhancements of ~ 39%, 21%, and 40% in PE, CE, and EQE, respectively, while 10% WS_2_ demonstrated a PE of 30.1 lm/W, a CE of 20 cd/A, and an EQE of 13.1%, representing enhancements of ~ 28% in PE, 20% in CE, and 35% in EQE. These findings underscore the superior efficacy of MoS_2_ as a dopant for hole injection layers in OLED applications. MoS_2_ outperforms WS_2_ as a dopant for hole injection layers mainly due to its superior hole mobility and optimized energy levels, which enhance charge injection efficiency and transport in OLED devices. Its elevated triplet energy level effectively minimizes energy losses, while the favorable alignment of HOMO and LUMO levels promotes efficient charge flow. In addition, the smoother surface of MoS_2_ enhances charge transport, leading to improved device efficiency. These advantages position MoS_2_ as a highly effective dopant for elevating the performance and longevity of OLED technology, demonstrating its potential for advancing the next generation of electroluminescent devices. Figure 4a–c illustrates the effects of doping on PEDOT:PSS at varying concentrations, highlighting the superior performance of the 10% MoS_2_ concentration at 100 cd/m^2^ compared to both PEDOT:PSS and WS_2_. The electroluminescence properties show that MoS_2_ significantly enhances power efficacy, current efficacy, and external quantum efficiency, underscoring its effectiveness as a hole injection layer in candlelight OLED devices. In Figure 4d, the luminance vs. voltage graph further supports these findings, with the inset image showcasing showing the 10% MoS_2_ device glowing brightly, highlighting its outstanding performance in practical applications.

### 2.3. Health and Environmental Considerations

**Figure 5:**
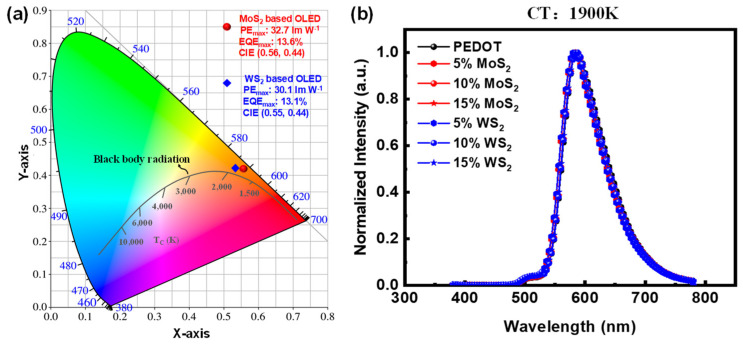
CIE coordinate diagram (**a**) illustrating the blackbody radiation curve, and (**b**) the normalized intensity vs. wavelength curve of the OLED device, with a color temperature of 1900 K, indicating alignment with natural light, which is beneficial for health and the environment.

The integration of 2D TMDs into candlelight OLED technology offers significant advantages in terms of health and environmental benefits. As shown in Figure 5a, the CIE coordinate diagram shows the alignment of the emission profile of the OLED device with the blackbody radiation curve at a color temperature of 1900 K.

The alignment of the OLED device’s emission profile with this blackbody curve is significant. The color rendering index (CRI) of the OLED device is 82, which indicates a light quality that is more aligned with warm, amber-like hues, similar to the color of candlelight. The chromaticity coordinates deviation (D_uv_) values for MoS_2_ and WS_2_ are 0.0097 and 0.0089, respectively, indicating a slight deviation above the blackbody curve, suggesting a chromaticity closer to the warm, amber-like hue of candlelight. It indicates that the light produced by the OLED is similar in quality and color to natural sunlight, enhancing overall light quality and creating a more inviting atmosphere. This close resemblance to natural lighting not only improves aesthetic appeal but also contributes positively to human well-being by fostering a soothing and comfortable environment.

Moreover, the warm light emitted can help mitigate melatonin suppression, promoting healthier sleep patterns compared to cooler, harsher light sources. Overall, the alignment depicted in Figure 5a underscores the potential of this OLED technology to create a lighting experience that supports both well-being and a more natural ambiance. In addition to this alignment, Figure 5b presents the normalized intensity vs. wavelength curve of the OLED device, further demonstrating its effective emission profile. The peaks in this curve highlight the alignment of the OLED light output with that of the blackbody radiation curve, reinforcing the benefits of using TMDs. This integration not only improves the efficiency of OLEDs by optimizing energy consumption and minimizing harmful emissions but also aligns with ESG goals. By promoting environmental sustainability through reduced energy consumption and waste, while addressing social concerns by enhancing user well-being through healthier lighting options, we contribute to broader sustainability initiatives. By prioritizing advanced materials such as 2D TMDs in candlelight OLED applications, we are advancing the technology and positively impacting both health and the environment.

## 3. Experimental Section

### 3.1. Instruments

Scanning electron microscopy (SEM) was conducted using two instruments, the FESEM JEOL 6500F was sourced from JEOL Ltd., Akishima, Japan and the Hitachi SU-8010 from Hitachi High-Tech, Tokyo, Japan, to analyze the surface morphology of MoS_2_ and WS_2_ nanostructures. Following this, ultraviolet photoelectron spectroscopy (UPS) analysis was performed with a U-3010 instrument from Hitachi High-Tech, Tokyo, Japan to evaluate the work function and highest occupied molecular orbital (HOMO) levels of these materials. UV-Vis absorbance measurements were then carried out using an HP-8453 diode array spectrometer from Agilent Technologies, Santa Clara, CA, USA, which operates within the wavelength range of 400 to 800 nm. Absorbance measurements were also taken with the same spectrometer, covering the full visible spectrum from 400 to 800 nm. In addition, X-ray diffraction (XRD) analysis was performed with a Bruker D2 Phaser utilizing Cu-Kα radiation (λ=1.54178 Å) to assess the crystallinity of MoS_2_ and WS_2._ Lastly, atomic force microscopy (AFM) measurements were conducted using a Bruker Dimension ICON to study the nanoscale topography.

### 3.2. Synthesis of TMDs (MoS_2_ and WS_2_)

For MoS_2_ and WS_2_ preparation, high-purity molybdenum (Mo, 99.99%) and tungsten (W, 99.99%) powders, along with sulfur (S, 99.95%), were obtained from Sigma-Aldrich, St. Louis, MI, USA and used as starting materials. Precise amounts of these powders were placed into a quartz tube, which was then sealed under a vacuum of $10^{-3}$ mbar. The tube was heated at a rate of 1.3 °C/min in a furnace up to 1000 °C and held at this temperature for 12 h, followed by slow cooling. The resulting material was ground into a fine powder and further annealed at 800 °C for 24 h to improve its crystallinity. To obtain a few layers of MoS_2_ and WS_2_ sheets [14], portions of the annealed powder were dispersed in solutions. For MoS_2_, a 10 mL solution containing 45% ethanol (EtOH) and 55% DI water was used. For WS_2_, a solution with 35% ethanol and 65% water was employed. The dispersions were sonicated for 8 h and then centrifuged at 3000 rpm for 20 min to remove larger aggregates, yielding a supernatant enriched with few-layer TMD sheets (Figure S4). The exfoliated few-layer MoS_2_ and WS_2_ were then utilized as hole injection layers (HILs) in OLEDs. To incorporate these TMDs, a specific concentration of the exfoliated MoS_2_ and WS_2_ in their respective dispersions was mixed with a Poly(3,4-ethylenedioxythiophene) polystyrene sulfonate (PEDOT: PSS).

### 3.3. Device Fabrication

Candlelight OLED devices were fabricated with the following configuration: ITO (~125 nm)/HIL (~35 nm)/EML (~10 nm)/TPBi (~50 nm)/LiF (~1 nm)/Al (~100 nm). ITO-coated glass substrates were first cleaned with a soap solution for 20 min, followed by rinsing with distilled water for 2 min. Later, the cleaned glass substrates were ultrasonically cleaned in acetone at 50 °C for 30 min and in isopropyl alcohol at 60 °C for another 30 min. After cleaning, the substrates were treated with ultraviolet ozone (UVO) light for 10–20 min to remove any residual moisture and then transferred to a nitrogen-filled glove box. The hole injection layer (HIL) was prepared by incorporating varying concentrations (5%, 10%, and 15%) of MoS_2_ and WS_2_ into a PEDOT:PSS solution, which was then spin-coated onto the ITO substrates at 4000 rpm for 20 s and annealed at 120 °C for 10 min. The emissive layer (EML) was fabricated by spin-coating a solution containing 10 wt% Ir(ppy)_3_ (green emitter) and 7.5 wt% Ir(2-phq)_3_ (orange-red dopant) in a TCTA host at 2500 rpm for 10 s. The substrates were positioned in a thermal evaporation system for the installation of additional layers, including the TPBi electron transport layer (ETL), LiF electron injection layer (EIL), and Al cathode. This process was performed at a base pressure of $4\times 10^{-6}$ Torr.

#### Test Conditions After Device Fabrication

After the OLED devices were fabricated, testing was performed in a controlled dark room to minimize the influence of extraneous light. A Keithley 2400 voltmeter from Keithley Instruments, Cleveland, OH, USA was used for current-voltage-luminance (J-V-L) measurements. The voltage was gradually increased from 0 to a predetermined maximum, allowing analysis of the current response and brightness of the devices. Stable temperature and humidity levels were maintained throughout the testing process to ensure accurate and reproducible results.

### 3.4. Characterizations

The devices were characterized using a CS-100A luminance meter for current density-voltage-luminance measurements and a Photo Research SpectraScan PR-655 spectrophotometer for evaluating current efficacy-luminance and power efficacy characteristics. All measurements were conducted in an artificial dark room to eliminate external light interference. Each device was tested with a consistent area of 0.09 cm^2^, ensuring accurate and comparable results. This comprehensive analysis provided valuable insights into the performance and efficiency of OLED devices.

## 4. Conclusions

In this study, we investigated the synthesis, characterization, and application of 2D TMDs, specifically MoS_2_ and WS_2_, in candlelight OLEDs. Our findings demonstrate that OLEDs doped with 10% MoS_2_ achieved significant improvements in performance metrics, including a power efficacy of 32.7 lm/W, a current efficacy of 21 cd/A, and an external quantum efficiency of 13.6%, representing enhancements of approximately 39%, 21%, and 40%, respectively, over the control device. In contrast, OLEDs with 10% WS_2_ exhibited a power efficacy of 30.1 lm/W, a current efficacy of 20 cd/A, and an external quantum efficiency of 13.1%, showing lower overall performance. The enhanced efficiency associated with MoS_2_ is due to its superior hole injection properties, optimized energy levels, and smoother surface morphology, which collectively facilitate more efficient charge transport and minimize energy losses. Future research should focus on the scalability of TMD synthesis and its integration into different OLED architectures to further enhance performance. In addition, exploration of other 2D materials may reveal new combinations that increase energy efficiency and meet ESG goals, ultimately contributing to sustainable and health-conscious lighting solutions.

## Figures and Tables

**Table 1:** AFM analysis of WS_2_ and MoS_2_ dopants in PEDOT:PSS is at 5%, 10%, and 15% compared to PEDOT:PSS.

Material Name	Doping Concentration (%)	R_a_ (nm)	R_q_ (nm)
PEDOT:PSS		0.695	1.030
MoS_2_	5%	0.438	0.558
	10%	0.410	0.523
	15%	0.427	0.552
WS_2_	5%	0.619	0.801
	10%	0.483	0.606
	15%	0.532	0.669

**Table 2:** Electroluminescence properties of the candlelight devices based on varying concentrations of dopants in the hole injection layer (HIL).

Dopant	Doping Concentration %	Driving Voltage (V)	Operation Voltage (V)	Power Efficacy (lm/W)	Current Efficacy (cd/A)	EQE (%)	CIE	Maximum Luminance (cd/m^2^)
			@ 100/1000/10,000 cd/m^2^					
PEDOT/PSS	0	2.8	3.5/4.3/6.1	23.6/17.5/9.8	25.7/26.8/19.2	9.7/9.8/6.6	(0.55, 0.44)/(0.55, 0.44)/(0.54, 0.45)	25,990
MoS_2_	5	2.6	3/3.8/6.4	28.6/23.4/9	29.1/29.3/18.2	12.9/12.1/8.8	(0.56, 0.44)/(0.55, 0.44)/(0.54, 0.45)	29,270
	10	2.6	3/3.8/5.8	32.7/25.6/11.6	31/31.4/21.5	13.6/13/9.3	(0.56, 0.44)/(0.55, 0.44)/(0.55, 0.45)	26,550
	15	2.6	3.1/3.9/6	30.4/23.6/10.8	30.1/28.9/20.7	13.1/12.8/9	(0.56, 0.44)/(0.55, 0.44)/(0.55, 0.45)	28,730
WS_2_	5	2.7	3.2/4.1/5.9	25.2/21.5/11.5	27.6/29.7/22.1	11.5/12.3/9.1	(0.55, 0.44)/(0.55, 0.44)/(0.54, 0.45)	26,330
	10	2.7	3.2/4.1/6.3	30.1/24.5/12.3	30.7/32.1/24.8	13.1/13.9/9.5	(0.55, 0.44)/(0.54, 0.44)/(0.53, 0.45)	27,150
	15	2.7	3.2/4.2/5.9	28.9/23.3/13	1.4/30.8/24.4	12.2/12.9/9.3	(0.55, 0.44)/(0.54, 0.44)/(0.54, 0.45)	30,320

## Data Availability

All data produced or examined in this study are provided within this published article.

## Supplementary Materials

The following supporting information can be downloaded at: https://www.mdpi.com/article/10.3390/molecules30010027/s1. Figure S1: Ultraviolet photoelectron spectroscopy (UPS) analysis of work function and highest occupied molecular orbital (HOMO) levels of WS_2_ and MoS_2_ flakes. Panels (a) and (b) show the work function measurements and corresponding HOMO levels for WS_2_, indicated in blue. Panels (c) and (d) show the same for MoS_2_ nanoparticles, highlighted in red. Figure S2: AFM image of the PEDOT:PSS control device, illustrating its surface morphology. Figure S3: UV-Vis spectra of MoS_2_ and WS_2_. Panels (a) and (b) show the absorption and optical bandgap measurements for MoS_2_, while panels (c) and (d) show the corresponding absorption and optical bandgap data for WS_2_. Figure S4: Flow chart illustrating the preparation and exfoliation processes of MoS_2_ and WS_2_. The chart details each step involved in the synthesis and recovery of monolayer materials from bulk precursors. Table S1: The table below shows the hole mobility values for each sample.
