# Supplementary material for: Two-Dimensional Transition Metal Dichalcogenide: Synthesis, Characterization, and Application in Candlelight OLED
Source: Molecules. 2024 Dec 25;30(1):27. doi: 10.3390/molecules30010027 (PMC11720818; doi:10.3390/molecules30010027)
Supplement: Supplementary file 1 [file molecules-30-00027-s001.zip › molecules-3324900-supplementary.pdf]

Supplementary Materials

Two-Dimensional Transition Metal Dichalcogenide: Synthesis, Characterization, and Application in Candlelight OLED

Dipanshu Sharma [1], Sanna Gull [1], Anbalagan Ramakrishnan [2], Sushanta Lenka [1], Anil Kumar [1], Krishan Kumar [3], Pin Kuan Lin [1], Ching-Wu Wang [4], Sinn-Wen Chen [2], Saulius Grigalevicius [5,*] and Jwo-Huei Jou [1,*]

[1] Department of Materials Science and Engineering, National Tsing Hua University, 101, Sec. 2, Guang-Fu Road, Hsinchu 30013, Taiwan; dipanshusharma7374@gmail.com (D.S.); sannagull@gapp.nthu.edu.tw (S.G.); sushantalenka1@gmail.com (S.L.); anilgpchkee@gmail.com (A.K.); kuan841216@gmail.com (P.K.L.)

[2] Department of Chemical Engineering, National Tsing Hua University, 101, Sec. 2, Guang-Fu Road, Hsinchu 30013, Taiwan; anbusrr@gmail.com (A.R.); swchen@mx.nthu.edu.tw (S.-W.C.)

[3] School of Chemical Sciences Indian Institute of Technology, Mandi 175005, Himachal Pradesh, India; krishanme906@gmail.com

[4] Graduate Institute of Opto-Mechatronics, Department of Mechanical Engineering, National Chung Cheng University, Chiayi County 62102, Taiwan; melcww@ccu.edu.tw

[5] Department of Polymer Chemistry and Technology, Kaunas University of Technology, Radvilenu Plentas 19, LT50254 Kaunas, Lithuania

* Correspondence: saulius.grigalevicius@ktu.lt (S.G.); jjou@mx.nthu.edu.tw (J.-H.J.)

1. Experimental Supplies

1.1. Materials

All chemicals used in this study were of analytical grade and employed directly without further purification. High-purity molybdenum (Mo, 99.99%) and tungsten (W, 99.99%) powders, along with sulfur (S, 99.95%) and poly(3,4-ethylenedioxythiophene)-poly(styrenesulfonate) (PEDOT:PSS), were obtained from Sigma-Aldrich. Tris(4-carbazoyl-9-ylphenyl) amine (TCTA), 2,2’,2” -(1,3,5-benzenetriyl)-tris(1-phenyl-1-H-benzimidazole) (TPBi), lithium fluoride (LiF), and the iridium complexes Tris(2-phenylpyridine) iridium ($Ir(ppy)_3$) and the orange-red emitter Ir(2-phq)$_3$ were sourced from Shine Materials. Filter paper was purchased from Merck Millipore.

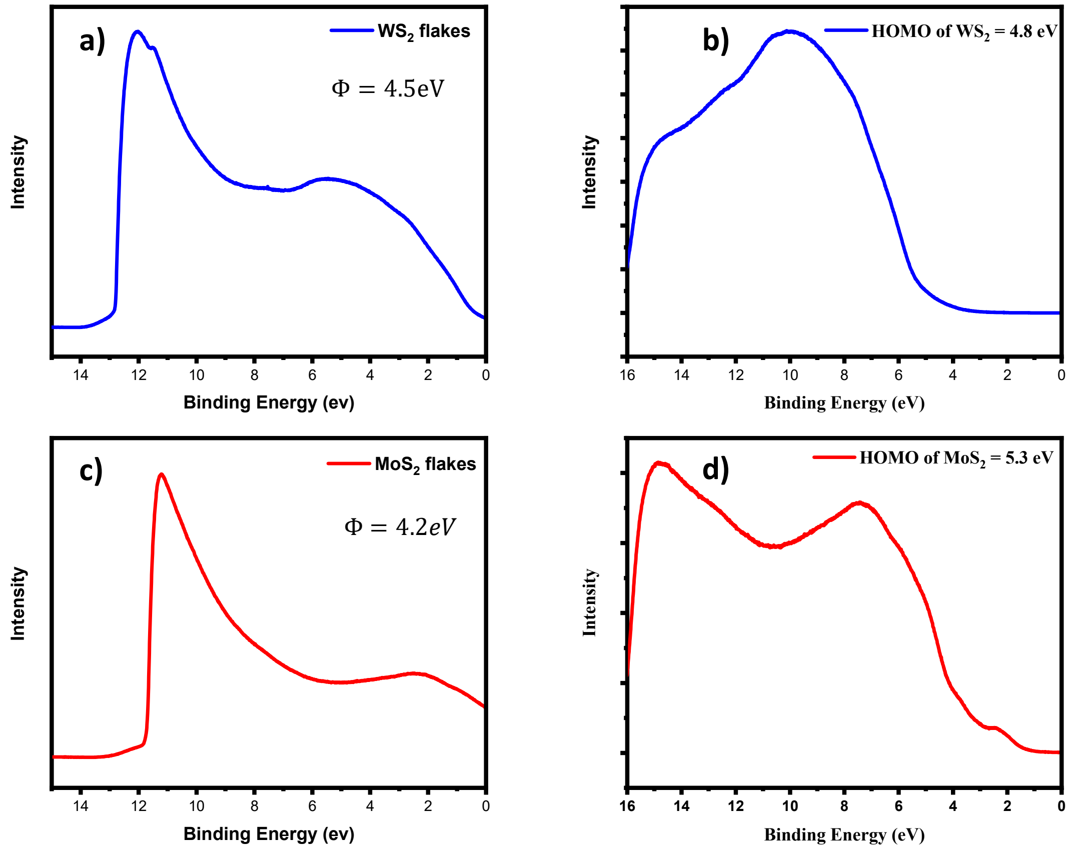

Figure S1. Ultraviolet photoelectron spectroscopy (UPS) analysis of work function and highest occupied molecular orbital (HOMO) levels of WS_2 and MoS_2 flakes. Panels (a) and (b) show the work function measurements and corresponding HOMO levels for WS_2, indicated in blue. Panels (c) and (d) show the same for MoS_2 nanoparticles, highlighted in red.

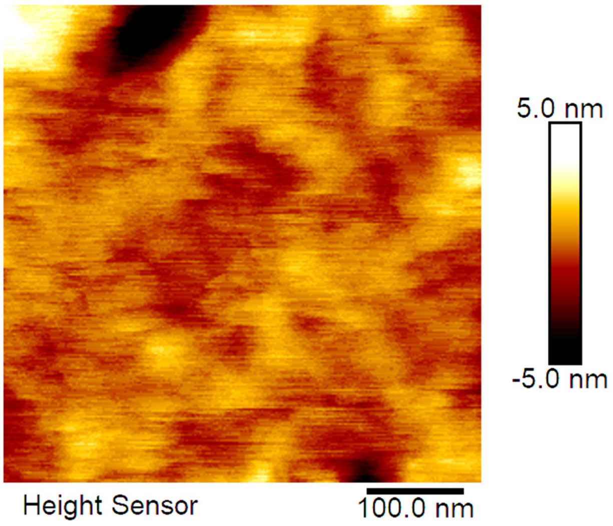

Figure S2. AFM image of the PEDOT:PSS control device, illustrating its surface morphology.

The space-charge-limited current (SCLC) method was used to determine the hole mobility of the samples. The SCLC for organic materials can be expressed by the Mott–Gurney (MG) equation:

$$\mu = \frac{8J\,d^3}{9\varepsilon_0\varepsilon_r v^2}$$

where μ is the hole mobility, J is the current density, d is the active layer thickness, ε_r is the relative permittivity (with a value of 3), and ε_0 is the permittivity of free space (with a value of 8.85×10^{-12} F/m). The calculated hole mobility values for each sample are presented in **Table S1** below.

Table S1. The table below shows the hole mobility values for each sample.

**No.**	**Sample**	**μ (cm^2/V·s)**
1.	PEDOT:PSS	2.3×10^{-4}
2.	MoS_2 5%	3.52×10^{-4}
3.	MoS_2 10%	4.95×10^{-4}
4.	MoS_2 15%	3.86×10^{-4}

**5.**	WS_2 5%	3.4×10^{-4}
**6.**	WS_2 10%	3.76×10^{-4}
**7.**	WS_2 15%	3.68×10^{-4}

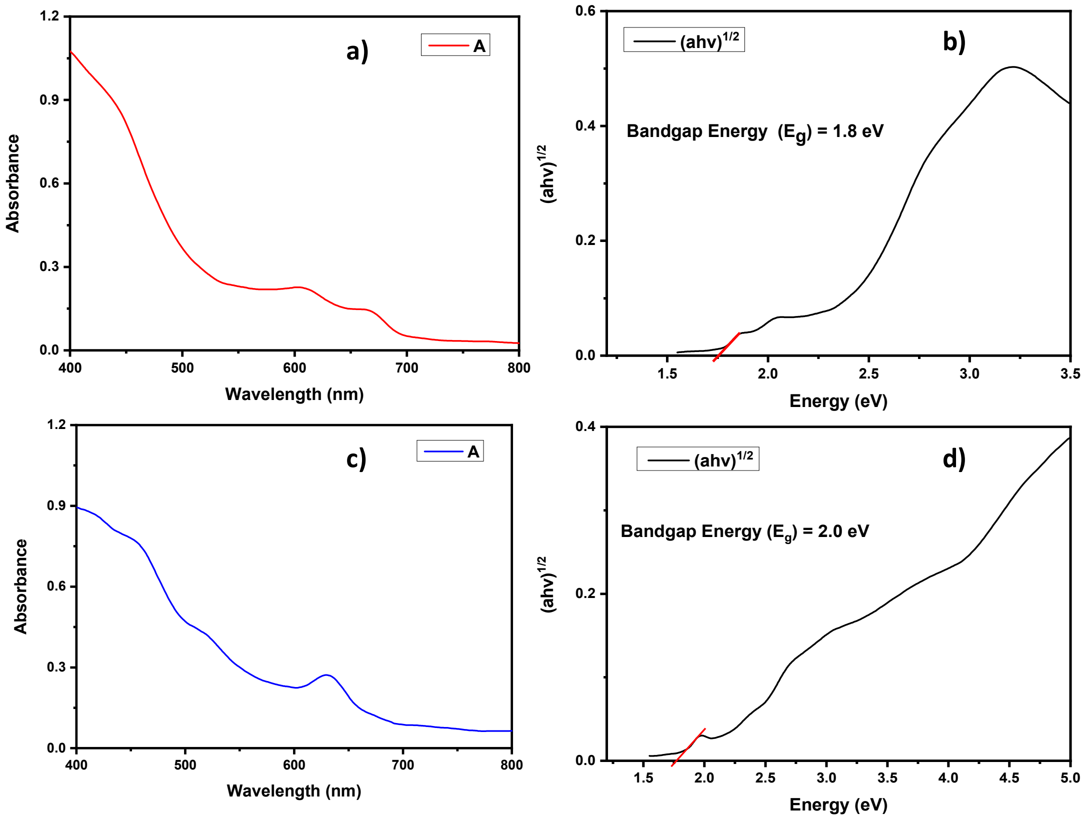

Figure S3. UV-Vis spectra of MoS_2 and WS_2. Panels (a) and (b) show the absorption and optical bandgap measurements for MoS_2, while panels (c) and (d) show the corresponding absorption and optical bandgap data for WS_2.

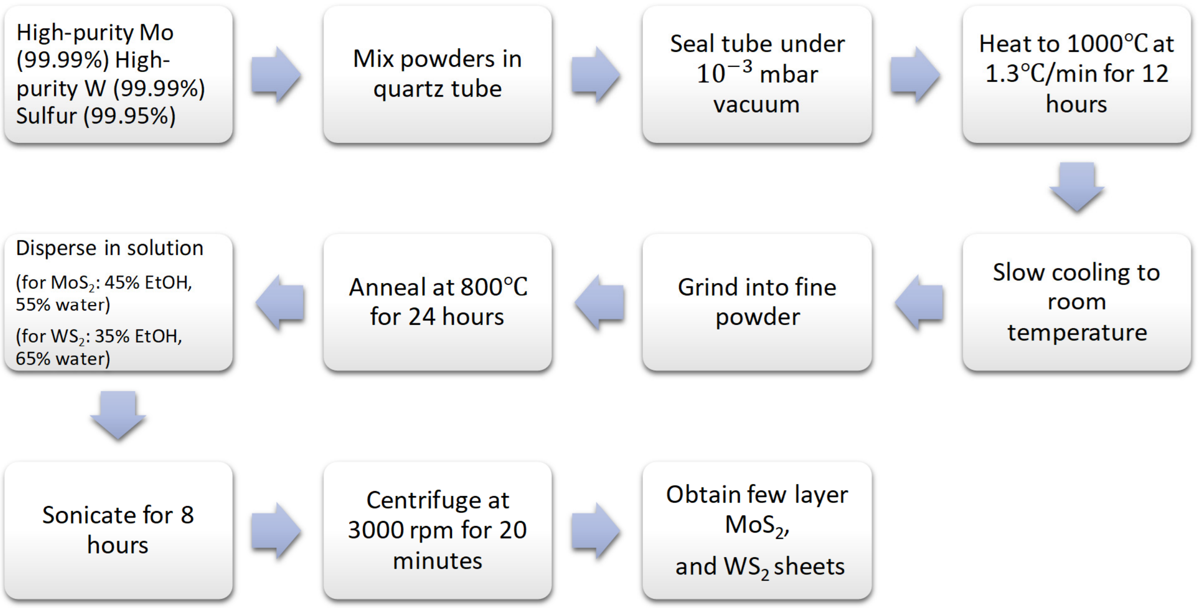

Figure S4. Flow chart illustrating the preparation and exfoliation processes of MoS_2 and WS_2. The chart details each step involved in the synthesis and recovery of monolayer materials from bulk precursors.
